# Ketosis-Prone Diabetes (Flatbush Diabetes): an Emerging Worldwide Clinically Important Entity

**DOI:** 10.1007/s11892-018-1075-4

**Published:** 2018-10-02

**Authors:** Harold E. Lebovitz, Mary Ann Banerji

**Affiliations:** Division of Endocrinology, Department of Medicine, State University of New York Health Science Center at Brooklyn, 450 Clarkson Ave., Box 1205, Brooklyn, NY 11203 USA

**Keywords:** Ketoacidosis, Type 2 diabetes mellitus, Minority populations, Treatment of severe hyperglycemia, Remission of diabetes, Insulin dependence, Insulin independence, Flatbush diabetes

## Abstract

**Purpose of Review:**

Ketosis-prone diabetes or Flatbush diabetes has been widely recognized as a clinical entity since 1984. Most of the early clinical studies focused on African American or Afro-Caribbean individuals. It is now being recognized as an important clinical entity in sub-Saharan Africans, Asian and Indian populations, and Hispanic populations. Major questions remain as to its pathogenesis and whether it is a unique type of diabetes or a subset of more severe type 2 diabetes with greater loss of insulin action in target tissues. This review summarizes the main clinical and mechanistic studies to improve the understanding of ketosis-prone (Flatbush) diabetes.

**Recent Findings:**

Little data are available on the magnitude of KPD in the different susceptible populations. It is relatively common in black populations. KPD is defined as a syndrome in which diabetes commences with ketoacidosis in individuals who are GAD and anti-islet cell antibody negative and have no known precipitating causes. The patients present during middle age, are overweight or mildly obese, and in many reports are more likely to be male. After intensive initial insulin therapy, many patients become insulin independent and can be well controlled on diet alone or diet plus oral medications.

**Summary:**

The clinical course of KPD is like that of patients with type 2 diabetes rather than that of type 1 diabetes. Little differences are found in the clinical characteristics and clinical outcomes between patients presenting with KPD and those presenting with severe hyperglycemia with no ketoacidosis. The mechanisms responsible for the development of ketosis-prone diabetes as well its remission remain unknown.

## Overview and Introduction

It has been recognized since the 1980s that some patients presenting with diabetic ketoacidosis may have a clinical course more like that of patients with type 2 diabetes than that of patients with type 1 diabetes. Winter et al. described a form of atypical maturity-onset diabetes of youth in 12 of 129 young black Americans [1•]. These patients initially were thought to have type 1 diabetes since several presented with ketosis, and all required initial insulin therapy. After periods of weeks to months, they were no longer dependent on insulin. This atypical diabetes occurred in two generations in 9 of 12 families and was not associated with islet-cell auto-antibodies. The recognition that adult black Americans with type 2 diabetes could present initially with diabetic ketoacidosis with no known precipitating cause (19 of 21 patients) was described by Banerji et al. [2•] under the rubric “Flatbush diabetes.” This syndrome was characterized by the acute onset of severe hyperglycemia with ketoacidosis requiring hospital admission and treatment with insulin and fluid and electrolyte replacement. After several weeks to months, 12 of 21 patients no longer required insulin and could be treated with diet alone or diet plus oral agents. All patients were GAD (glutamic acid decarboxylase) and anti-islet cell antibody negative. An increase in frequencies of HLA DR3 and DR4 compared to non-diabetic black control patients was found. This syndrome has been named ketosis-prone type 2 diabetes (KPD) and now has been documented in all non-Caucasian populations in which it has been sought. This includes black populations (African American, African-Caribbean, sub-Saharan African) [2••, 3•, 4••, 5••], Hispanic populations [6], and Asian (Chinese, Indian, and Japanese) [7–10] populations. Table 1 lists some of the major studies in which KPD has been reported and characterized.

**Table 1:** Ketosis-prone diabetes (KPD) has been reported to occur many non-Caucasian populations including those of African background (Brooklyn, Atlanta, and sub-Saharan countries), Hispanic populations (Houston), and Asian populations (China, India). A summary of salient features from these disparate populations provides an analysis of the constant features which comprise this syndrome

Population (reference)	Brooklyn [2]	Atlanta [4]	Africa [5]	Houston [6]	Shanghai [10]	India [9]
Number (KPD/total population)	21/21	35	111	51/103 DKA	18/238 < 35 years	11/34 DKA
Age (years)	M 40.8 ± 9.8 F 51.1 ± 6.3	40 ± 2	39.1 ± 9.5	39 ± 12	28.2 ± 4.8	39.8 ± 6.5
Male/female	12/9	25/10	84/27	1.7/1	8/1	8/3
Body mass index (kg/m^2^)	M 27.8 ± 2.7 F 30.0 ± 4.1	Ideal body weight 157 ± 6%	28.5 ± 5.1	29.4 ± 8.3	28 ± 4.5	25.3 ± 1.6
Race						
Black	21	35	111	14 (27 %)		
Hispanic				30 (59%)		
Chinese					18	
Indian						11
Family history of DM (number)	14	29	75	45	13	7
New-onset DM (number)	19	25	111	26	14	11
DKA number (pH)	21 (7.18 ± 0.09)	35 (7.25 ± 0.10)	66	51 (< 7.30)	9/18 (≤ 7.30)	11 (7.14 ± 0.08)
Ketosis number	0	0	45	NA	9/18 pH > 7.30	0
Admission glucose (mmol/l)	38.5 ± 11.6	38 ± 2	30.5 ± 5.1	26.5 ± 10.4	10.4 ± 3.4	32.6 ± 7.7
Admission HbA1c (%)		12.8 ± 0.4	13.4 ± 2.1	13.8 ± 2.5	11.9 ± 1.5	11.3 ± 1.8
Fasting C-peptide (ng/ml)	1.7 ± 1.0	1.5 ± 0.1	NA	1.94 ± 0.13	1.6 ± 0.7	0.46 ± 0.08
Stimulated C-peptide (ng/ml)	4.6 ± 3.4	2.5 ± 0.2	1.0 1.3	19.52 ± 1.35 (0–10 min)	2.8 ± 1.1	1.02 ± 0.1
GAD; ICA auto-antibodies	negative	negative	negative	negative	negative	negative
HLA	Increase DR3 & DR4		No Association DRB-1;DRQ1			
Initial treatment with insulin (number)	21	35	111	51	17/18 < 4 months	11/11
Insulin requiring at follow-up (number)	9	10/35	27 (24%)	25	0/18	0/11
Remission (no therapy) (number)	6	NA	NA	5	11/18	0
Remission (diet + oral meds) (number)	6	NA	84 (76%)	21	7/18	11
HbA1c (%) during remission	5.7 ± 1.6	6.8 ± 0.2	*IR 6.9 ± 0.3 *NIR 5.6 ± 0.1	7.5 ± 2.1	5.6 to 7.1 (14)	6.1 ± 0.3

*NIR* non-insulin treated remission, *IR* insulin treated during remission, *DM* diabetes mellitus, *DKA* diabetic ketoacidosis, *HbA1c* hemoglobin A1c, *GAD* glutamic acid decarboxylase antibody, *ICA* islet cell antibody, *HLA* human leucocyte antigen

From these publications, a clinical entity can be defined, although as will be discussed later, there are some differences that exist among the various populations reported. The patient with ketosis-prone type 2 diabetes is more often male, middle-aged, overweight, or modestly obese (type 1 obesity); has a family history of type 2 diabetes; presents with new-onset severe hyperglycemia and ketosis or frank diabetic ketoacidosis; and is GAD and islet cell antibody negative. They require initial treatment with insulin and fluid and electrolyte replacement. Following several weeks to months of insulin treatment, their metabolic abnormalities improve, and they may be managed by diet alone or diet plus oral antidiabetic agents. A small percentage may continue to require insulin therapy. Recurrent ketoacidosis is unusual, and the clinical course is like that of a patient with type 2 diabetes.

Many questions exist about the entity of ketosis-prone diabetes. What are the pathogenic mechanisms involved in its development? Is it a unique type of diabetes mellitus? How should its clinical course be managed?

## Pathogenic Mechanisms

In assessing the mechanisms responsible for ketosis-prone diabetes, there are two fundamental questions. The first is, are there a unique set of circumstances that lead to acute disruption of metabolic regulation leading to severe hyperglycemia and ketoacidosis that resolve by restoration of euglycemia? The second is, do patients who develop ketosis-prone diabetes have unique abnormalities of beta cell function and/or insulin resistance that are part of their metabolic regulatory systems. Obviously, the first question can be addressed by acute studies at or shortly after presentation of ketosis-prone diabetes. The second question is addressed by evaluating the metabolic state of ketosis-prone diabetic patients after the acute event has resolved and during long-term near-normoglycemic follow-up.

The acute abnormalities of ketosis-prone diabetes have been examined using intravenous glucose tolerance tests, oral glucose tolerance tests, and intravenous glucagon stimulation tests [3•, 6, 11]. Table 2 summarizes the data on insulin secretion in ketosis-prone diabetes. The beta cells of patients with severe hyperglycemia with or without ketosis have lost the ability of exogenous or endogenous glucose to stimulate β-cell insulin secretion. The ability of glucose to stimulate insulin secretion begins to return after 2 weeks of normoglycemic treatment and maximizes by 8 to 12 weeks of normoglycemic treatment. In contrast, there is modest although impaired insulin secretion in response to pharmacologic doses of intravenous glucagon within days after resolution of the ketoacidosis. This response increases with time but remains less than that of ordinary obese type 2 diabetic patients. In long-term follow-up, patients with ketosis-prone diabetes separate into those who continue to require insulin treatment and those who can be treated to near-normoglycemia with diet alone or diet plus oral agents. Those continuing to require insulin have a severe decrease in stimulated insulin secretion while those not requiring insulin have only a modest reduction in stimulated insulin secretion [6, 12].

**Table 2:** Insulin secretory studies in ketosis-prone diabetes

Population studied (reference)	Atlanta [4]		Brooklyn [2, 3, 12]	Houston [6]	Sub-Sahara (5)	
	Acute studies (performed within days or week or two of resolution of DKA)					
Type of patient studied	Obese KPD	Obese T2DM	KPD		KPD Insulin dependent	KPD non-insulin dependent
	1 day after DKA and hyperglycemia treated		After DKA and hyperglycemia corrected	After DKA and hyperglycemia corrected	After initial treatment of DKA and hyperglycemia	
Plasma glucose (mmol/l)	11.5 ± 1	10.5 ± 1	N/A	N/A	6.16 ± 1.7	6.82 ± 2.3
Plasma insulin or C-peptide response						
GTT						
Fasting plasma insulin (μU/ml)	13	25	N/A	N/A	N/A	N/A
Mean 0–20 min plasma insulin (μU/ml)	13 ± 0.4	21 ± 0.1	N/A	N/A	N/A	N/A
IV glucagon						
Basal plasma C-peptide (ng/ml)	1.5	2.0	N/A	N/A	N/A	N/A
Stimulated plasma C-peptide) (ng/ml)	2.4	3.2	N/A	C-peptide area 0 to 10 min 19.5 ± 1.4	Increase above basal 0.9	Increase above basal 1.3
	Chronic studies (performed weeks or months after resolution of DKA)					
	12 weeks after DKA treated		4 to 120 months after DKA treated	6 months after DKA treated	12 months after DKA treated	
IV-GTT						
Fasting plasma insulin (μU/ml)	13	28	N/A	N/A	N/A	N/A
Mean 0–20 min plasma insulin (μU/ml)	59	48	N/A	N/A	N/A	N/A
Oral GTT						
Fasting C-peptide (ng/ml)	N/A	N/A	1.7 ± 1.04	N/A	N/A	N/A
Maximal C-peptide (ng/ml)	N/A	N/A	4.6 ± 3.4	N/A	N/A	N/A
IV glucagon						
Basal plasma C-peptide (ng/ml)	1.9	2.6	N/A	N/A	N/A	N/A
Stimulated plasma C-peptide) (ng/ml)	4.0	3.9	N/A	C-peptide area 0 to 10 min 32.9 ± 2.0	Increase above basal 1.1	Increase above basal 2.8

Insulin secretion was assessed immediately after normoglycemia was achieved (acute studies) or after some weeks or months of near-normoglycemia (chronic studies). Insulin secretion was assessed by the response to either IV or oral glucose or to IV glucagon. Measurement of insulin secretion was estimated by either plasma insulin or plasma C-peptide levels

*KPD* ketosis-prone diabetes. Data are mean ± SEM

*IV-GTT* intravenous glucose tolerance 0–20 min, *OGTT* oral glucose tolerance 0 to 120 min; IV glucagon 1 mg intravenously at time 0 with measurements to 10 min

Both alpha and beta cell function were evaluated in 15 sub-Saharan Africans with ketosis-prone diabetes (KPD) who were insulin free and normoglycemic and 15 matched normal control patients by Choukem and colleagues in Paris [13]. Fasting plasma glucose and insulin were higher; however, fasting plasma glucagon was not significantly different between the patients with KPD and the control patients. In response to an oral glucose challenge, early insulin secretion was markedly decreased, 2-h plasma insulin was increased, and 2-h plasma glucagon was the same between KPD patients and normal controls. An arginine stimulation test showed that KPD patients had markedly diminished insulin and C-peptide secretion, but similar glucagon responses when compared to the control population. During a euglycemic insulin clamp, there was no difference in baseline glucagon levels and no difference in glucagon suppression during the clamp between KPD patients and the normal controls. The studies did show that glucagon suppression relative to hyperglycemia was impaired as is characteristic of type 2 diabetes, but that basal and stimulated levels were not different from normal controls.

Umpierrez et al. assessed the possibility that patients with ketosis-prone diabetes might have an increased sensitivity to lipotoxicity [14]. After patients had achieved near-normoglycemic control off of insulin (approximately 12 weeks), the effect of 48-h intravenous infusions of 20% intralipid on beta cell function (plasma insulin and C-peptide levels) throughout the infusions were measured. There was no difference in the beta cell response in patients with ketosis-prone diabetes as compared to obese patients who had presented with severe hyperglycemia or non-diabetic obese controls. The insulin secretory responses to arginine infusions were likewise unaffected by the intravenous intralipid in all three cohorts.

Insulin resistance was assessed shortly after resolution of ketoacidosis by the frequently sampled intravenous glucose tolerance test, insulin tolerance test, and by HOMA-IR [6, 11, 15]. These measurements as well as the euglycemic hyperinsulinemic clamp were used to assess insulin resistance during long-term follow-up of patients with KPD. Table 3 summarizes these data. Insulin resistance is quite severe when measured within a few days of the initial event, and in the Atlanta and sub-Saharan black populations, it lessens over several weeks of euglycemic treatment after which it was only slightly increased in chronic follow-up. In contrast, insulin resistance remained severe in long-term follow-up of the black Brooklyn KPD and obese patients presenting with severe hyperglycemia [2••, 12].

**Table 3:** Studies measuring insulin resistance in patients with ketosis-prone diabetes

Population studied (reference)	Atlanta [4]			Brooklyn [2, 12]		Sub-Sahara [5, 13]			
	Acute								
	KPD	Obese T2DM	Obese non-diabetic controls	KPD	Normal controls	KPD-insulin-dependent	KPD-non insulin-dependent	Type 2 Diabetes	Normal Controls
	1 day after DKA treated					After initial treatment			
Plasma glucose (mmol/l)	11.5 ± 1	10.5 ± 1	5.3 ± 0.1	NA	NA	6.16 ± 1.7	6.82 ± 2.3	NA	NA
Insulin sensitivity									
IV-GTT (μU/ml/min)	0.3 ± 0.1	0.4 ± 0.1	1.3 ± 0.3						
Insulin tolerance test (%/min)						1.8 (*N* = 8)	1.6 (*N* = 12)	2.3 (*N* = 19)	4.8 (*N* = 8)
	Chronic								
	12 weeks			3 to 120 months		6 months			
Plasma glucose (mmol/l)	NA	NA	NA	5.9 ± 0.28 (*N* = 20)	5.6 ± 0.17	6.8 ± 1.4	NA	NA	5.2 ± 0.6
HbA1c (%)	6.8 ± 0.2	7.0 ± 0.2	NA	5.7 ± 1.6					
Insulin sensitivity									
IV-GTT (μU/ml/min)	1.2 ± 0.4	0.8 ± 0.3							
Euglycemic hyperinsulinemic clamp (mg/kg/min)				3.53 ± 0.4 (*N* = 20)	7.59 ± 0.4 (*N* = 9)	7.3 ± 3.0 (*N* = 15)	N/A	N/A	10.3 ± 3.8 (*N* = 15)
Insulin tolerance test (%/min)						2.5 (*N* = 8)	4.3 (*N* = 12)	2.8 (*N* = 19)	N/A

*KPD* ketosis-prone diabetes, *IV GTT* intravenous glucose tolerance test

In summary, the causes of the development of severe hyperglycemia in either overweight or obese patients with ketosis-prone diabetes or in the obese non-ketosis-prone patient with type 2 diabetes remain unknown. The diabetes is newly diagnosed in these patients at the time of their admission to the hospital for insulin treatment. The inability of glucose to stimulate insulin secretion appears to be the central abnormality leading to the severe hyperglycemia with or without ketoacidosis and persists for several days to weeks despite normalization of the plasma glucose. The ability of non-glycemic pharmacologic agents (glucagon and arginine) to stimulate insulin secretion during the acute phase indicates that even during the acute hyperglycemia, there is a small store of insulin within the beta cell which is not physiologically available but can be used to differentiate these patients from patients with classical type 1 diabetes.

## Is Ketosis-Prone Diabetes a Separate Subtype of Diabetes Mellitus?

Controversy exists as to whether ketosis-prone diabetes should be classified as a separate subtype of diabetes [16]. The literature supports two severe forms of type 2 diabetes presentations: one with acute severe hyperglycemia [3•, 5••, 12, 17•] and one with new-onset ketoacidosis with severe hyperglycemia [2••, 4••, 5••, 6]. Are they separate entities or are they one entity with a continuum of decreased cellular insulin action? Maldonado et al. have dissected ketosis-prone diabetes based on the magnitude of acute glucagon-stimulated insulin secretion and the presence or absence of diabetes-related auto-antibodies [6]. Patients with adequate beta cell function after short-term treatment and the absence of auto-antibodies is defined as the novel phenotype (ketosis-prone diabetes). Other investigators have viewed ketosis-prone type 2 diabetes as a more severe form of type 2 diabetes in which the available insulin action at the cellular level is markedly impaired acutely but is partially reversible with appropriate treatment [2••, 4••, 5••, 12].

If ketosis-prone diabetes is a separate entity, its characteristics other than marked ketosis should be different from those of new-onset severe hyperglycemia without ketosis. We have previously reported in separate publications the characteristics of these two populations of black diabetic patients presenting with new-onset severe hyperglycemia. Table 4 compares the characteristics of these patients: one with severe hyperglycemia and one with ketoacidosis and severe hyperglycemia. Except for the severe ketosis and acidosis, the populations are quite similar: middle age, male predominance, overweight or mildly obese, new-onset diabetes, extremely high-presenting plasma glucoses, requirement for initial insulin and fluid treatment, remission after weeks or months of euglycemia in a significant number of patients, and markedly impaired glucose-mediated insulin secretion which returns toward normal after euglycemic treatment. Severe insulin resistance was present even after euglycemia in 20/21 Brooklyn patients presenting with ketoacidosis. Our data in a black Afro-American, Afro-Caribbean population suggest that KPD is a similar although more insulin-deficient population than the severe hyperglycemic population without ketoacidosis. The available data in other black populations are consistent with our data. Mauvais-Jarvis and colleagues studied patients with both ketosis and ketoacidosis and assessed the long-term follow-up of two sub-Saharan black populations. They found that during chronic follow-up of patients with KPD, 25% remained insulin-dependent and 75% became insulin-independent patients. Patients who were insulin independent could became insulin dependent if they gained significant weight [5••]. Comparing Cameroonian patients in the non-ketotic phase of KPD to ordinary patients with type 2 diabetes, Lontchi-Yimagou and colleagues concluded that KPD in black African patients “is likely to be a subtype of type 2 diabetes with the potential to develop an acute insulinopenic phase at diagnosis” [15].

**Table 4:** Comparison of characteristics of black patients with type 2 diabetes presenting with ketosis-prone diabetes and those presenting with severe hyperglycemia [2, 3]

	Ketosis-prone diabetes (severe hyperglycemia and ketoacidosis)	Severe hyperglycemia	Normal controls
Number	21	26	16
Age (years)	M 40.8 ± 9.8 F 51.1 ± 6.3	48.8 ± 10.8	43.8 ± 8.8
Sex (M/F)	12/9	16/10	N/A
BMI (kg/m^2^)	M 27.8 ± 2.7 F 30.0 ± 4.1	28.5 ± 3.8	25.2 ± 1.0
Family history of diabetes	14 (67%)	18 (69%)	0
New-onset diabetes	19	26	N/A
Plasma glucose (mmol/l) at admission	38.5 ± 11.6	31.0 ± 12.8	5.2 ± 1.7
Ketoacidosis at admission	21 (pH 7.18 ± 0.09)	1	
Initial fasting plasma C-peptide (ng/ml)	N/A	1.47	
Initial Stimulated C-peptide (ng.ml^−1^ .min^−1^) AUC 0–120 min	N/A	271	
HbA1c (%) at 2 to 8 weeks	N/A	9.5 ± 0.6	
GAD and IC antibody	0/21	0/1	
Initial insulin treatment	21	26	
Normoglycemic remission	6/21 (28.6%) (FPG mmol/l = 6.3 ± 0.6)	11/26 (42.3%) (FPG mmol/l = 6.8 ± 0.43)	
Mean time to remission	9.5 months	83 days	
HbA1c at remission (%)	N/A	6.2 ± 0.2	
Treatment with insulin	9	9	
Treatment with oral agents	6	6	
HbA1c at time of study (%)	5.7 ± 1.6	7.1 ± 0.4	
Fasting C-peptide at follow-up (ng/ml)	1.7 ± 1.04	Remission 1.98 ± 0.18 No remission 2.13 ± 0.21	1.5 ± 0.5
Stimulated C-Peptide at follow-up (ng.ml^−1^ .min^−1^) AUC 0–120 min	393.2 ± 268.2	Remission 636.3 ± 81.0 No remission 471.8 ± 54.6	513.0 ± 120

*AUC* area under the curve during oral glucose tolerance test, *GAD* glutamic acid decarboxylase, *IC* islet cell, *FPG* fasting plasma glucose

## Could Ketone Metabolism Be Different in KPD

Ketones are a normal metabolic fuel whose generation and utilization are directly related to a decrease in the availability and utilization of glucose [18–21]. Ketogenesis requires an increase in adipose tissue lipolysis with transport of glycerol and free fatty acids to the liver [18, 19]. Glucagon stimulates, and insulin inhibits beta-oxidation which occurs primarily, though not exclusively, in hepatocyte mitochondria [18, 21]. Long chain fatty acids require carnitine palmitoyltransferase 1 to enter the hepatocyte mitochondria where beta-oxidation generates Acetyl-CoA. Acetyl-CoA molecules condense to form Acetylacetyl CoA which then adds an additional Acetyl-CoA to form 3-Hydroxy-3-methylglytaryl CoA (HMG-CoA). HMG-CoA is cleaved by the rate-limiting enzyme HMG-CoA lyase to acetoacetate which is processed to acetone or beta-hydroxybutyrate. While ketone production is primarily limited to the hepatocyte, ketolysis occurs in most peripheral tissues. Beta-hydroxybutyrate enters peripheral cells through monocarboxylate transporter 1 and is processed by succinyl-CoA: oxoacid CoA transferase (SCOT) and acetoacetyl CoA1-thiolase (ACAT-1) to Acetyl-CoA which generates energy through the tricarboxylic acid (TCA) cycle or forms lipids.

Elevations of plasma ketones in humans can occur by overproduction but is usually due to decreased ketolysis. A decrease in insulin accompanied by an increase in glucagon action in adipose tissue and liver is ordinarily the cause of increased ketogenesis which serves to increase ketones for utilization in energy-starved peripheral tissues. When ketolysis is impaired by deficient SCOT, ACAT-1, or an impairment in TCA cycle activity, hyperketonemia and or ketoacidosis develops.

Utilizing metabolomic approaches and then quantifying metabolic fluxes using in vivo stable isotope tracers, Patel et al. studied stored plasma samples from 20 obese, stable KPD patients collected 4 to 8 weeks after their index episode of ketoacidosis and compared the data to that of 19 obese non-diabetic control subjects [22]. Utilizing metabolomic differences identified in fatty acid, ketone, and amino acid pathways, they quantified those pathways in 9 newly recruited KPD patients who were on stable insulin therapy 6 to 8 weeks beyond their index ketoacidosis event and on 7 matched non-diabetic control subjects [22]. The results showed that KPD patients had decreased rates of release of peripheral fatty acids and their conversion to β-hydroxybutyrate compared to the non-diabetic controls. This indicated that the ketosis is due to decreased ketolysis. The major abnormalities found were accelerated leucine catabolism and transamination of α-ketoglutarate to glutamate, with impaired tricarboxylic cycle anaplerosis of glutamate carbon. These studies were done weeks after the index ketoacidosis and were compared to control non-diabetic patients rather than patients with type 2 diabetes or patients having presented with severe hyperglycemia without ketoacidosis. While interesting, Patel’s data fail to give a clear insight into the underlying pathogenesis of ketosis-prone diabetes. An excellent analysis of Patel’s data is in the accompanying editorial by Romas-Roman et al. [23].

## Clinical Relevance and Conclusions

The recognition and understanding of ketosis-prone diabetes is of considerable importance to clinicians. It is now recognized that many patients presenting with diabetic ketoacidosis, particularly if they are non-Caucasians, may have an atypical form of type 2 diabetes and not type 1 diabetes. These patients require hospitalization with insulin and fluid and electrolyte replacement. A significant percentage of these patients become insulin independent after several weeks to several months of insulin treatment and their glycemia can be managed with ordinary diet alone (remission) or diet plus oral agents for many years. Sulfonylurea therapy has been shown to prolong remissions in patients with ketosis-prone and acute severe hyperglycemia presentations [24, 25]. There are some differences in the syndrome depending on the racial or ethnic background of the population. Most of our current information comes from African background populations. However, recognition of the syndrome has resulted in increasing reports of the syndrome in Asian populations (Chinese, Japanese, Korean), Asian Indian populations, and Hispanic populations. The mechanisms for the acute onset of severe hyperglycemia with or without ketosis and ketoacidosis in susceptible patients are currently unknown. The impact of newer treatments such as sodium-glucose transporter 2 inhibitors (SGLT-2 i) which predispose to ketoacidosis needs to be evaluated before being administered to patients with known ketosis-prone diabetes.
